# How long should athletes with high range of motion demands stretch? Acute stretching durations for flexibility and performance: a systematic review

**DOI:** 10.3389/fphys.2026.1881773

**Published:** 2026-07-17

**Authors:** Ismail Belli, Mohammad Alghosi, Morteza Bagheri Kalayeh, Erfan Abdollahzadeh, Fatemeh Heidari, Ramila Abedi Azar, Mohammad Alimoradi, Mojtaba Iranmanesh, Elham Hosseini, Andreas Konrad

**Affiliations:** 1 Department of Coaching Education, Faculty of Sports Sciences, Haliç University, Istanbul, Türkiye; 2 Department of Physical Education, Technical and Vocational University (TVU), Tehran, Iran; 3 Department of Exercise Physiology, Faculty of Physical Education and Sport Sciences, University of Tehran, Tehran, Iran; 4 Department of Sports Injury and Corrective Movements, Faculty of Physical Education and Sport Sciences, University of Tehran, Kish International, Tehran, Iran; 5 Department of Sport Sciences, Imam Reza International University, Mashhad, Iran; 6 Laboratory for Robotic Research, Iran University of Science and Technology, Tehran, Iran; 7 Department of Sports Injuries and Corrective Exercises, Faculty of Sports Sciences, Shahid Bahonar University of Kerman, Kerman, Iran; 8 HERC- Health, Exercise & Research Center, Dubai, United Arab Emirates; 9 Institute of Human Movement Science, Sport and Health, Graz University, Graz, Austria

**Keywords:** athletic preparation, flexibility enhancement, muscle performance, range of motion optimization, stretching protocols

## Abstract

**Background:**

Stretching is a common practice among athletes in sports requiring high flexibility, but the optimal duration and type of stretching to enhance performance while minimizing potential drawbacks remains unclear.

**Objective:**

This systematic review aims to evaluate the acute effects of different stretching durations and techniques—specifically static, dynamic, and combined methods—on flexibility and performance in athletes engaged in flexibility-dependent sports.

**Methods:**

Following PRISMA guidelines, a systematic search of PubMed, Web of Science, and Scopus was conducted up to March 2026. Twenty-three studies met the inclusion criteria, encompassing 627 athletes from sports such as gymnastics, swimming, wrestling, dance, and track and field. Data were synthesized using thematic analysis and percentage-weighted mean changes with 95% confidence intervals, comparing pre- to post-intervention outcomes or intervention groups to controls (depending on study design). Moderating variables such as stretching type and duration were also considered.

**Results:**

Static stretching produced moderate improvements in flexibility from pre- to post-intervention (2.97%) and a moderate improvement when compared to control (2.36%), but was associated with small negative performance effects pre to post (−0.88%) and trivial declines compared to control (−0.07%). Dynamic stretching showed small flexibility gains (0.77%) compared to control, and small improvements in performance (0.55%). Combined protocols resulted in small positive effects on both flexibility (1.65%) and performance (0.60%). PNF interventions produced a small positive effect on performance (1.04%) when compared to control conditions. Regarding duration, short-duration stretching (≤60 s) led to a small flexibility improvement compared to control (0.79%), with trivial performance gains pre to post (0.06%) and compared to control (0.48%). Long-duration stretching (>60 s) yielded large flexibility improvements pre to post (6.21%) and a small improvement compared to control (1.89%), but small performance declines pre to post (−1.80%) and trivial effects relative to control (−0.02%). However, the majority of included studies were rated as low quality (15/23), and findings should be interpreted with caution.

**Conclusion:**

Stretching duration affects flexibility and performance. Long static stretching boosts flexibility but slightly reduces performance, while dynamic stretching enhances readiness with minimal flexibility gains. Protocols should match sport-specific demands and session goals.

**Systematic review registration:**

https://www.crd.york.ac.uk/PROSPERO/view/, identfier CRD42025635493.

## Highlights

- Static stretching increases range of motion (ROM) but can temporarily reduce performance, making it better suited for dedicated flexibility sessions rather than pre-competition warm-ups, especially in athletes with high ROM requirements.
- Dynamic stretching is may be preferred for warm-ups, as it can enhance performance, although it results in small gains in flexibility when compared to static stretching. This makes dynamic stretching a practical option for athletes who need both mobility and power output, such as those with high ROM demands.
- Stretch duration is important:

- Short-duration stretching (≤ 60 s) helps preserve performance while offering small flexibility benefits.
- Long-duration stretching (> 60 s) maximizes ROM gains but can negatively affect performance.
- For athletes with high ROM needs, program design should balance flexibility goals with performance outcomes.

## 1 Introduction

Flexibility is essential for athletes, enabling them to perform movements with greater range and efficiency (Iranmanesh et al., 2025). As individuals age, however, the deterioration of muscles and connective tissues leads to a decline in both strength and flexibility, limiting functional capacity. Research has indicated a progressive reduction in flexibility across age groups from 20 to 49 years (Halder et al., 2015), with an average decrease of about 10% every decade. This decline can significantly impact daily activities and diminish the quality of life for adults (Stathokostas et al., 2012). In this respect, stretching is a fundamental component of athletic training and has been advocated since the early 1980s as a method to improve athletic performance (Shrier, 2005; Rudisill et al., 2023).

To improve flexibility, stretching is most commonly employed, and its effectiveness is believed to be duration-dependent (Warneke et al., 2023). Recommendations for stretching exercises are regularly updated, highlighting the importance of duration in achieving optimal flexibility and performance outcomes (Babault et al., 2021). While brief static stretches lasting less than 30 s may not substantially hinder performance, durations between 30 to 45 s typically have minimal impact, and stretches exceeding 60 s can lead to significant decreases in muscle strength and power (Kay and Blazevich, 2012; Behm et al., 2016; Bogdanis et al., 2019; Warneke and Lohmann, 2024). This dose-response relationship suggests that static stretching (SS) lasting 60 s or longer leads to greater performance deficits than shorter durations, in contrast to dynamic stretching (DS), which improves performance. This is consistent with the finding that SS temporarily impairs neuromuscular activation, but incorporating sports-specific dynamic movements afterward can mitigate these losses by restoring activation and reducing stiffness-related effects (Behm et al., 2016).

Current guidelines suggest incorporating DS into warm-up routines to maximize performance benefits while minimizing potential drawbacks (Blazevich et al., 2018; Babault et al., 2021; Afonso et al., 2024). However, there remains a gap in the understanding of the precise impact of different stretching durations on athletic performance, necessitating further research (Afonso et al., 2024).

The conclusion drawn, i.e., static stretches exceeding 60 s likely reduce muscle performance, is based on a population with and without flexibility requirements. Athletes in sports such as gymnastics, dance, swimming, track and field, and wrestling require exceptional range of motion (ROM) to excel (Kapo et al., 2016; Sands et al., 2016; Ruggieri and Costa, 2019; Franchini and Herrera-Valenzuela, 2021; Haddad et al., 2021). Consequently, incorporating stretching exercises into training regimens can be a crucial strategy for enhancing flexibility and optimizing performance, particularly in these sports (McNeal and Sands, 2006; Lima et al., 2019). Hence, there is a need to establish if such thresholds (i.e., 60 s) and specific stretching technique recommendations (dynamic > static) are also relevant for athletes with high flexibility demands, or if there is a difference with the general population.

Hence, given the nature of these sports with high flexibility demands, as well as the variability in study findings and methodologies regarding stretching techniques, a systematic review is essential to synthesize the existing research on acute stretching durations in this specific population. This comprehensive evaluation will clarify the effects of different stretching protocols and provide clearer recommendations for athletes and coaches (Blazevich et al., 2018; Chaabene et al., 2019). This systematic review aims to evaluate the acute effects of different stretching durations and techniques, including static, dynamic, proprioceptive neuromuscular facilitation (PNF), and combined stretching methods, on flexibility and performance in athletes engaged in flexibility-dependent sports.

## 2 Methods

This systematic review was conducted following the guidelines outlined in the Preferred Reporting Items for Systematic Reviews and Meta-Analyses (PRISMA) statement (Page et al., 2021) and has been registered with the International Prospective Register of Systematic Reviews (PROSPERO) under registration number CRD42025635493.

### 2.1 Search strategy

A comprehensive search of original articles was conducted, covering publications up to March 1, 2026, across three international electronic databases: Web of Science, PubMed, and Scopus. The searches were independently executed by two authors—M.ALG. and M.ALI.—with any discrepancies addressed through discussion and, if necessary, the input of a third author, M.BK. The search strategy utilized MeSH terms along with relevant text words. The complete search strategy employed the Boolean method: ((stretch*) AND (acute OR single OR immediate* OR sudden) AND (gymnastics* OR swim* OR diving* OR “martial art” OR skate* OR skiing* OR dance* OR climb* OR wrestle* OR cheerlead* OR “track and field”)). Specific search strings can be found in **Supplementary Table 1**. This review imposed no language restrictions, and Google Translate was employed to interpret non-English studies. In addition, the reference lists of the included studies were manually screened, and a grey literature search was conducted covering conference proceedings, dissertations, clinical trial registries, and preprint platforms such as medRxiv and bioRxiv. The identified studies were organized using Endnote, and duplicate entries were eliminated. The search for relevant research was further supplemented using the Connected Papers website (https://www.connectedpapers.com/).

### 2.2 Eligibility criteria

The Population, Intervention, Comparison, Outcomes, and Study (PICOS) framework (Methley et al., 2014), along with the inclusion and exclusion criteria, is detailed in **Table 1**.

**Table 1:** Eligibility criteria based on the PICOS framework.

PICOS element	Inclusion criteria	Exclusion criteria
Population	Athletes participating in sports where above-average flexibility and large joint ROM are important for performance, technical execution, or aesthetics., including gymnastics, swimming, diving, martial arts, skating, skiing, dance, climbing, wrestling, cheerleading, and track and field (pole vault and high jump).	Athletes from sports other than those in the inclusion criteria. History of lower extremity or spinal surgery. Non-human studies.
Intervention	Studies that explored the immediate effects of various stretching techniques, including static, dynamic, ballistic, PNF, and other forms of stretching.	Multiple interventions compared simultaneously, incorporating combined approaches such as stretching paired with other exercise interventions, excluding warm-ups.
Comparison	Studies that primarily compared stretching protocols (static, dynamic, ballistic, or PNF) to a control condition (no stretching/placebo). Studies without control groups (e.g., single-group designs or direct comparisons between stretching techniques) were included only for secondary analyses of technique or duration effects, provided that their data were analyzed separately and explicitly labeled as uncontrolled evidence, to maintain the primary synthesis integrity for the controlled comparisons.	Absence of pre-and post-comparisons.
Outcome	Studies that measured outcomes related to flexibility (e.g., ROM) and performance (e.g., sprint speed, vertical jump height, muscle strength, balance, competitive scores and benchmarks in gymnastics, swimming, diving, martial arts, skating, skiing, dance, climbing, wrestling, cheerleading, and track and field).	Parameters other than those in the inclusion criteria as outcome variables. Studies with insufficient or unclear data on stretching duration or protocol.
Study design	RCTs and non-RCTs (including quasi-experimental studies with control groups, or pre- to post-comparisons without control groups).	Case studies, reviews, studies with insufficient or unclear data on stretching duration or protocol.

non-RCT, non-randomized controlled trial; RCT, randomized controlled trial; PNF, proprioceptive neuromuscular facilitation.

### 2.3 Study selection

The selection of relevant articles was conducted by two independent authors—M.ALG. and M.ALI.—with prior experience in systematic reviews. Initially, all the studies retrieved from the databases were imported into EndNote reference library (version 21; Clarivate Analytics, Thomson Reuters Corporation, Philadelphia, Pennsylvania), where duplicate entries were eliminated. The titles and abstracts were then independently assessed by both researchers and, if necessary, the full papers were also evaluated. The eligibility criteria were applied, and in cases of disagreement, a third person was consulted to resolve the disagreement (A.K.). This process was similarly applied during the full-text screening of the remaining articles, to make the final decisions. Finally, the reference lists of the included articles were examined to identify any additional studies that may have been overlooked in the initial search; however, no further articles were found for inclusion.

### 2.4 Data extraction

The extraction process was carried out by two researchers (E.H. and E.A.) and subsequently verified by two independent researchers (F.H. and M.I.). The extracted items included the following: authors, study design, sample size, age, participant sport, intervention, outcomes, and key findings.

### 2.5 Data synthesis

Due to the high heterogeneity resulting from the diverse types of sports, athletes, and stretching frequency, intensity, time, and type (FITT), a meta-analysis was not conducted. Instead, a thematic analysis approach was utilized to identify the key themes and patterns across the included studies. The primary outcome measures focused on the performance and flexibility of athletes. In addition, several moderating variables were considered when synthesizing the findings, including the type of stretching and stretching duration. The subsequent sections detail the percentage-weighted mean changes from pre- to post-intervention and between intervention and control groups, with 95% confidence intervals. Percentage changes were calculated as [(post - pre)/pre × 100], weighted by sample size. Given the heterogeneity of outcome types, these values are presented descriptively to illustrate patterns, not as pooled effect estimates. In line with previous recommendations, we categorized the calculated percentage-weighted mean changes into distinct magnitudes: changes below 0.5% were classified as trivial, those between 0.5% and less than 2% as small, changes from 2% to less than 5% as moderate, from 5% to less than 10% as large, and changes exceeding 10% as very large (Behm et al., 2016). These magnitude thresholds are adopted from prior stretching research (Behm et al., 2016) for descriptive purposes only. The practical significance of a given percentage change may vary across outcomes; therefore, thresholds should not be interpreted as equivalent across all measures. In addition, prior to data merging, agreement between reviewers during the literature search process was systematically assessed using kappa (κ) statistics. The strength of agreement was categorized into distinct levels: poor (κ ≤ 0.20), fair (κ = 0.21–0.40), moderate (κ = 0.41–0.60), substantial (κ = 0.61–0.80), and near-perfect (κ = 0.81–0.99) (Cohen, 1968).

### 2.6 Quality assessment

The Downs and Black checklist (Downs and Black, 1998) is widely regarded as the most suitable tool for assessing the quality of both randomized controlled trials (RCTs) and non-randomized controlled trials (non-RCTs) (Deeks et al., 2003; Alzahrani et al., 2019). For this study, a 15-item modified version of the checklist, as adapted by Zadro et al., was utilized (Zadro et al., 2017). The scoring criteria followed a binary system: “yes” responses received 1 point, while “no” or “unable to determine” responses were assigned 0 points. An exception was made for item 4, which employed a three-tiered scoring system (2 points for “yes”, 1 for “partially”, and 0 for “no”). The maximum possible score was 16, with a predefined exclusion threshold set at ≤8 points (50% threshold), based on established methodological standards. The overall quality was categorized as follows: low (≤10 points), moderate (11 or 12 points), and high (≥13 points) (Chen et al., 2009; Munn et al., 2010). The evaluations were independently conducted by two authors (M.B.K. and R.AA.), and any discrepancies were resolved through consultation with a third author (M.ALI.).

### 2.7 Levels of evidence

The levels of evidence, as defined by the Oxford Centre for Evidence-Based Medicine (OCEBM) (Howick et al., 2011), were assigned to each study by one author (M.ALI.) and subsequently verified by a second reviewer (M.ALG.). The OCEBM framework ranks evidence according to study design and susceptibility to bias, with lower numerical levels indicating stronger evidence (Howick et al., 2011). In general, Level II studies provide stronger evidence and a lower risk of bias than Level III studies.

## 3 Results

### 3.1 Search results

**Figure 1:**
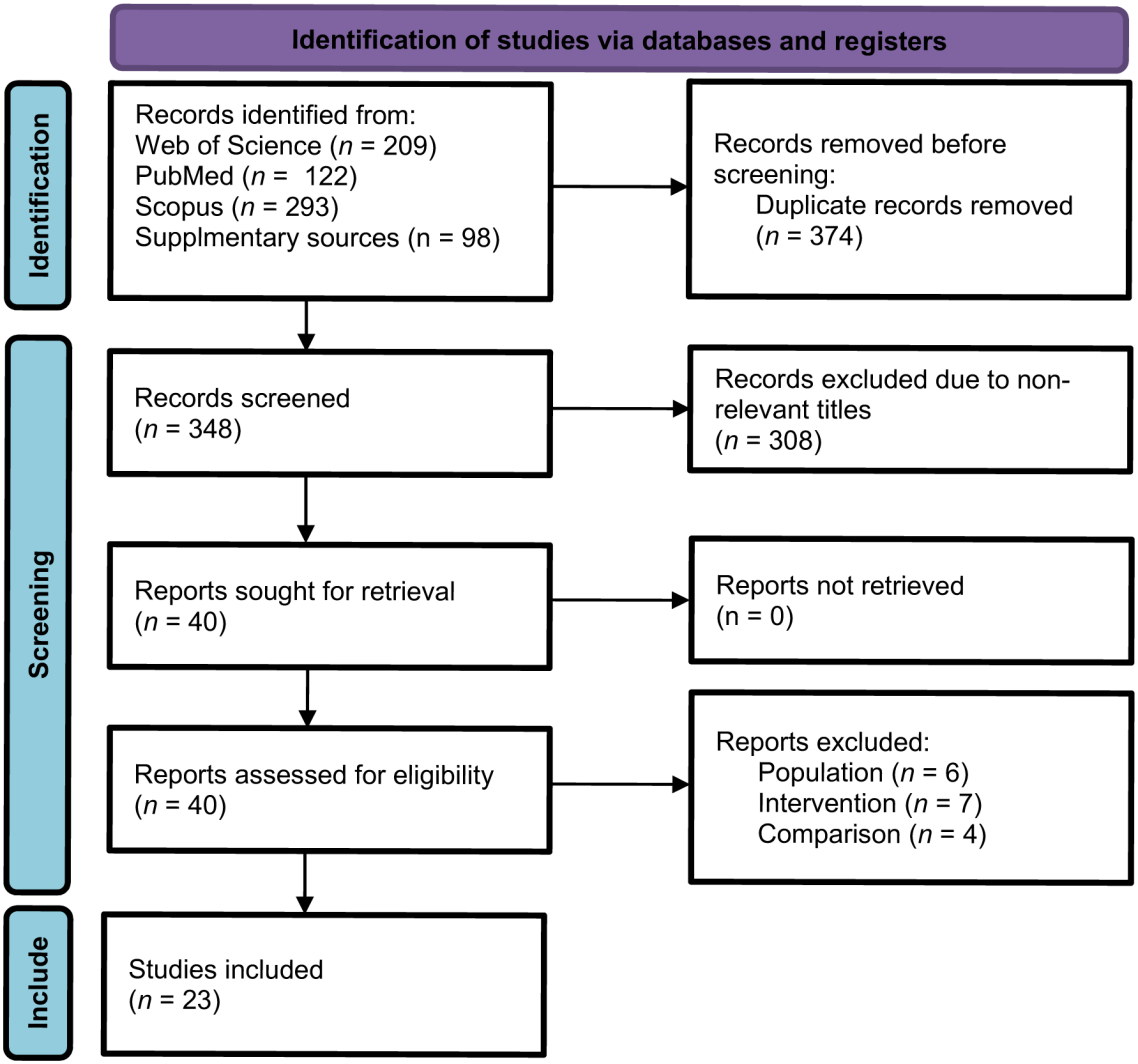
Preferred Reporting Items for Systematic Reviews and Meta-Analyses (PRISMA) 2020 flow diagram for new systematic reviews, including searches of databases and register.

The search initially identified 722 articles from the electronic databases. A total of 374 duplicate articles were removed, and 308 articles were excluded. In total, 40 articles were screened for eligibility. A total of 17 articles were excluded due to not meeting inclusion criteria (**Supplementary Table 2**). Supplementary sources (reference lists, grey literature including conference proceedings, dissertations, trial registries, and preprints, and the Connected Papers website) provided 98 records. After screening using the same eligibility criteria, no additional studies beyond the 23 database-derived studies were included (**Figure 1**). The reviewers’ kappa value for the final articles was 1.0 (perfect agreement; **Supplementary Table 3**). The included studies included 627 (min–max: 8–74) participants with a mean age of 19.6 ± 4.8 years (range: 9.8–28.5 years). Among the 580 participants with sex reported, 339 (58.4%) were females and 241 (41.6%) were males. One study (Yan et al., 2025) with 24 participants did not report sex. The participant population consisted of athletes involved in various sports, including swimming, gymnastics, skiing, dance, and wrestling. The characteristics and interventions of the included studies are summarized in **Table 2**, while the key findings are presented in **Table 3**.

**Table 2:** Study characteristics and interventions of included studies.

Authors	Study design	Sample size	Age (years)	Participant sport	Intervention	Outcomes
1. Sands et al., 2008	RCT	11; F: 11	20.6 ± 2.5	Swimming	Single session Duration: 40 s Protocol: SS (forward split stretching for 40 s) hip flexors and thigh (rear leg)	Passive forward split ROM Active forward split ROM
2. Di Cagno et al., 2010	Non-RCT	38; F:38	14.1 ± 3.2	Gymnastics	Single session Duration: 10 min Protocol: SS (3 × 30 s) hamstrings, calves, back extensors	Vertical jump test (squat jump, CMJ, hopping test) Gymnastics-specific split leaps test
3. Rubini et al., 2011	RCT	45 (EG: 30 CG: 15); F:45	28.5 ± 8.0	Ballet	Single session Duration: ~4–5 min Protocol: SS (4 × 30 s)/PNF (4 × 10-10–10 s) hip adductors	Hip flexibility assessment
4. Agopyan et al., 2012	Non-RCT	29; F:14 M:15	11.6 ± 0.4	Swimming	Single session Duration: NR Protocol: SS (2 × 30 s) quadriceps, iliopsoas, hamstrings, gastrocnemius, gluteus maximus, tibialis anterior, plantar foot muscles	25 and 50-m short-distance flutter kicking performance tests
5. Williams et al., 2013	Non-RCT	29; F:25 M:4	19.5 ± 1.2	Swimming	Single session Duration: ~5–10 min Protocol: PS (2 × 30 s) pectoralis minor	Pectoralis minor length
6. Agopyan et al., 2013	Non-RCT	26; F:20 M:6	18.2 ± 2.1	Dance	Single session Duration: ~5–7 min Protocol: SS (3 × 30 s) hamstrings	Knee flexion and extension isokinetic test
7. Morrin and Redding, 2013	Non-RCT	10; F:10	27.0 ± 5.0	Dance	Single session Duration: ~8–10 min Protocol: SS (2 × 30 s), DS (2 × 30 s), CS (15 s SS + 15 s DS) quadriceps, hamstrings, gastrocnemius, gluteus maximus	VJ test Balance Hamstring ROM
8. Cengiz et al., 2014	Non-RCT	15; M:15	23.2 ± 2.6	Wrestling	Single session Duration: ~3 min Protocol: SS (6 × 30 s), DS (6 × 5 slow + 10 fast reps) hamstrings, quadriceps, calves, biceps, triceps, shoulders	Wingate test performance
9. Silva et al., 2014	Non-RCT	13; M:13	22.7 ± 1.4	Swimming	Single session Duration: ~5 min Protocol: SS (2 × 30 s), PNF-3S (2 × 30 s) quadriceps	50-m front crawl swimming test
10. Donti et al., 2014	Non-RCT	34; F:24 M:10	20.3 ± 3.0	Gymnastics	Single session Duration: ~5–10 min Protocol: SS (15 s)/SS (30 s) quadriceps, hamstrings, calves	Straight leg raise ROM CMJ
11. Bogdanis et al., 2019	Non-RCT	16; M:16	24.0 ± 4.0	Gymnastics	Single session Duration: ~5 min Protocol: ISS (3 × 30 s)/CSS (1 × 90 s) quadriceps and hip flexors	Modified Thomas test Single-leg CMJ
12. Johnson et al., 2018	Non-RCT	27; F:27	11.5 ± 1.7	Gymnastics	Single session Duration: ~7 min Protocol: SS (4 × 30 s) hip flexors, quadriceps, hamstrings	Dynamic flexibility Jump height
13. Papia et al., 2018	Non-RCT	19; F:19	9.8 ± 0.5	Gymnastics	Single session Duration: ~4 min Protocol: SS (1 × 90 s) quadriceps and hip flexors	One-leg CMJ Two-leg CMJ Hip joint ROM Knee joint ROM
14. de la Cruz-Torres et al., 2019	RCT	45 (stretching: 15 eccentric: 15 PNM: 15); F: 45	20.0 ± 3.4	Ballet	Single session Duration: ~2–3 min Protocol: SS (4 × 20 s) flexor hallucis longus	ROM of the first metatarsophalangeal joint Balance
15. Dallas et al., 2019	Non-RCT	26; F:26	22.4 ± 3.6	Gymnastics	Single session Duration: ~5–10 min Protocol: DS (10 s, 20 s, 30 s, 40 s) quadriceps, hamstrings, gluteus maximus, plantar flexors	20-m sprint run T-test (agility test)
16. Balcı et al., 2020	RCT	74 (Neural sliding: 38 Neural stretching: 36); F:15 M: 59	17.9 ± 2.3	Wrestling	Single session Duration: ~6 min Protocol: NS (3 × 60 s)/NSG (3 × 60 s) hamstrings	AKEL SR test
17. Pessali-Marques et al., 2020	Non-RCT	46; M:46	24.5 ± 0.8	Dance	Single session Duration: ~3 min Protocol: CTS (6 × 30 s) hamstrings	Maximal ROM
18. Dierick et al., 2021	Non-RCT	16; F:12 M:4	15.3 ± 2.8	Dance	Single session Duration: ~2–5 min Protocol: SS (2 × 30 s)/DS (10 × 10 s) hamstrings	Kinematics of grand battement: (e.g., angle, velocity, etc.) Esthetics of grand battement: scored by a jury of professional dancers
19. Arı, 2021	Non-RCT	8; F:8	15.3 ± 1.0	Wrestling	Single session Duration: ~10–12 min Protocol: SS (30 s)/DS (30 s)/CSD and CDS (30 s alternating) hamstrings, quadriceps, upper-lower extremity	10-m sprint CMJ Medicine ball throw SR test
20. Kurt et al., 2024	Non-RCT	17; M:17	20.0 ± 4.0	Wrestling	Single session Duration: ~6.8–9.3 min Protocol: DS (slow tempo – 50 bpm/moderate tempo – 100 bpm/fast tempo – 120 bpm) lower body multi-muscle	Jump height Relative power Reactive strength index
21. Durukan et al., 2025	RCT	28; M:28	22.2 ± 1.9	Wrestling	Single session Duration: ~5–10 min Protocols: SS (11 exercises × 20-30s)/DS (11 exercises × 10-30s) full body	Static balance Dynamic balance
22. Jochum et al., 2025	RCT	8; M:8	17.9 ± 1.0	Skiing	Single session Duration: 20min Protocol: PS (2 × 2 min per side, except quadriceps: 1 × 2 min) hamstrings, quadriceps, adductors	ROM (hip abduction, hip flexion, passive SLR, passive knee extension, stand and reach)
23. Yan et al., 2025	RCT	24 (23 analyzed); Sex not reported	19.5 ± 0.7	Dance	Single session Duration: 20 min Protocols: PNF (3 × 30s), SS (3 × 30s), DS (3 × 30s; 20 reps/min) iliopsoas, quadriceps, gluteus maximus, hamstrings, gastrocnemius	SR test CMJ height Dynamic balance

AKEL, active knee extension limitation; CG, control group; CMJ, countermovement jump; CS, combined stretching; CSD, combined static + dynamic stretching; CDS, combined dynamic + static stretching; CSS, continuous static stretching; CTS, constant torque stretching; DS, dynamic stretching; EG, experimental group; F, female; ISS, intermittent static stretching; M, male; Non-RCT, non-randomized controlled trial; NS, neural stretching; NSG, neural stretching group; PNF, proprioceptive neuromuscular facilitation; PNM, ultrasound-guided percutaneous neuromodulation; PS, passive stretching; RCT, randomized controlled trial; ROM, range of motion; RPE, rating of perceived exertion; SLR, straight leg raise; SR, sit and reach; SS, static stretching.

**Table 3:** Key findings of included studies.

Authors	Key findings
1. Sands et al., 2008	↑ Passive forward split ROM (significantly increased with vibration + stretching compared to stretching only, p = 0.002) ↔ Active forward split ROM (no significant difference between vibration + stretching and stretching only, p = 0.21)
2. (Di Cagno et al., 2010	↔ VJ flight time (unaffected by SS warm-up, p > 0.05) ↓ Flight time (significantly reduced for split leap with leg stretched, split leap with ring, and split leap with back bend of the trunk after SS, p < 0.01) ↓ Judges’ scores (significantly decreased after SS, p < 0.001)
3. Rubini et al., 2011	↑ Flexibility (significantly improved post-stretching with both PNF and SS, p < 0.0001) ↔ Flexibility (no significant difference between PNF and SS, p = 0.235)
4. Agopyan et al., 2012	↔ Flutter kicking swim times (no significant difference between stretching and no-stretch conditions for 25 m and 50 m, p > 0.05)
5. Williams et al., 2013	↑ Pectoralis minor length (significantly increased with gross stretch compared to controls, p = 0.007)
6. Agopyan et al., 2013	↑ Isokinetic thigh strength: Significantly increased following SS in flexion (PT%BW and TW) at 180°s-1, p < 0.05. In extension, significant increase was observed at 180°s-1 in TW and at 300°s-1 in PT%BW and TW, p < 0.05 ↓ Isokinetic thigh strength: significant decrease in extension at 60°s-1 in PT and PT%WB, p < 0.05. ↔ No significant difference was observed in other strength parameters in flexion and extension at different angular velocities, p > 0.05.
7. Morrin and Redding, 2013	↑ VJ height, balance, and ROM (significantly improved with CS compared to SS, p < 0.05) ↑, ↓ VJ height and ROM (significantly improved and decreased, respectively, between DS and SS, p < 0.05) ↓ ROM (significantly decreased between DS and control, p < 0.05) ↔ VJ height, balance, and ROM (no significant difference between SS vs. control, DS vs. control, and CS vs. control, p > 0.05)
8. Cengiz et al., 2014	↓ Power (significantly decreased with DS compared to SS)
9. Silva et al., 2014	↓ 50-m front crawl performance (significantly reduced with SS and PNF compared to control, p < 0.0001) ↔ SS vs. PNF (no significant difference between SS and PNF, p > 0.05)
10. Donti et al., 2014	↑ ROM (increased significantly after short stretching compared to rest condition, p < 0.01) ↑ ROM (increased significantly after long stretching duration compared to rest condition, p < 0.01) ↔ CMJ (no significant changes after both stretches compared to baseline, p > 0.05)
11. Bogdanis et al., 2019	↓ CMJ height (decreased significantly immediately after continuous stretching, p = 0.001) ↓ CMJ height (decreased significantly 1 min post-continuous stretching, p = 0.001) ↑ Hip joint ROM (increased significantly with intermittent stretching and with continuous stretching, p = 0.001) ↑ Knee joint ROM (increased significantly with intermittent stretching and with continuous stretching, p = 0.001)
12. Johnson et al., 2018	↓ Split jump flexibility (significantly decreased, p < 0.05) ↔ Jump height: No significant effect observed, p > 0.05.
13. Papia et al., 2018	↔ One-leg CMJ height, no significant effects for time, leg, or interaction, p = 0.278, p = 0.207, p = 0.444 ↔ Two-leg CMJ height (no significant change, p = 0.186) ↑ Hip joint ROM (increased after stretching, p = 0.002) ↔ Knee joint ROM (no significant change, p = 0.218)
14. de la Cruz-Torres et al., 2019	↑ ROM (significantly improved for the flexor hallucis longus muscle, p = 0.009) ↔ Balance (no significant effect, p > 0.05)
15. Dallas et al., 2019	↑ Sprint performance (significantly improved in 20-m sprint after 20 s (p < 0.001) and 30 s (p < 0.005) of DS) ↑ Agility performance (significantly improved after 20 s (p < 0.001), 30 s (p < 0.005), and 40 s (p < 0.05) of DS)
16. Balcı et al., 2020	↔ AKEL (no significant differences between the two techniques for AKEL in both legs, p > 0.05) ↑ AKEL (both techniques showed a significant improvement in AKEL for both the right leg (p = 0.001) and left leg (p = 0.001) from pre- to post-mobilization) ↑ SR test (both techniques showed a significant improvement in flexibility (p = 0.001) from pre- to post-mobilization)
17. Pessali-Marques et al., 2020	↑ Dancers exhibited a significant increase in [ROM.sub.Max], p < 0.05)
18. Dierick et al., 2021	↑ ROM (DS significantly improved the thigh ROM compared to the control condition, p = 0.007) ↔ ROM (no significant effect was observed between SS and control condition, p = 0.069) ↔ Aesthetic quality (no significant changes in the aesthetic quality of movements, as assessed by a jury, across all conditions, p > 0.05)
19. Arı, 2021	↑ Speed (DS (p = 0.050) and CSD (p = 0.043) significantly improved speed compared to the CG ↓ Speed (SS group showed significantly slower speed than control, p = 0.012) ↑ VJ (DS (p = 0.041) and CSD (p = 0.043) significantly improved VJ compared to the control) ↔ VJ (no significant effect was observed between SS and control condition, p > 0.05) ↔ Flexibility (no significant differences in flexibility, p > 0.05) ↔ Medicine ball throwing (no significant differences, p > 0.05)
20. Kurt et al., 2024	↔ Jump performance (no significant differences in maximum jump height, minimum jump height, mean jump height, relative power, and reactive strength index across DS conditions, p > 0.05)
21. Durukan et al., 2025	↑ Dynamic balance (significant difference in favor of DS group, d = 0.91, p = 0.023). ↑ Freestyle wrestlers had higher dynamic balance than Greco-Roman after both DS (d = 1.88, p = 0.008) and SS (d = 1.47, p = 0.022). ↑ Greco-Roman wrestlers had higher static balance than freestyle after both DS (d = 1.52, p = 0.018) and SS (d = 1.70, p = 0.014).
22. Jochum et al., 2025	↑ ROM (PS significantly increased ROM compared to CG, p < 0.05).
23. Yan et al., 2025	↑ Flexibility: PNF (ES = 0.32, p<0.01), SS (ES = 0.26, p<0.01), and DS (ES = 0.30, p<0.01) all significantly improved SR compared to CG. ↔ Power and Balance: No significant changes in CMJ or dynamic balance for any stretching protocol (p > 0.05).

AKEL, active knee extension limitation; CG, control group; CMJ, countermovement jump; CS, combined stretching; CSD, combined static + dynamic stretching; DS, dynamic stretching; PNF, proprioceptive neuromuscular facilitation; PS, passive stretching; PT, peak torque; PT%BW, peak torque % body weight; ROM, range of motion; SR, sit and reach; SS, static stretching; TW, total work; ↑, significant increase; ↓, significant decrease; ↔, no change.

### 3.2 Quality assessment and level of evidence

The Downs and Black quality assessment revealed that, among the 23 studies, four (17.4%) were rated as high quality, four (17.4%) as moderate quality, and 15 (65.2%) as low quality. The mean score was 10.65; however, this mean should be interpreted with caution, as the majority of studies fell into the low-quality category, while only eight studies were rated as moderate or high quality. Thus, the overall body of evidence is best characterized as low to moderate, with a predominance of lower-quality studies. The lowest scores were due to unclear representation of the subjects asked to participate in the study and the question as to whether those who were willing to participate reflected the broader population. Conversely, the highest scores were attributed to clear description of the hypotheses, aims, and main outcomes, as well as well-defined participant characteristics. When reported, the distributions of principal confounders were presented transparently. The main findings were effectively articulated, and estimates of random variability were provided, with any data dredging clearly indicated. The statistical tests used were appropriate, and the outcome measures were shown to be accurate, valid, and reliable, ensuring that subjects in the different intervention groups were recruited from the same population. The results of the Downs and Black quality assessment for each study can be found in **Supplementary Table 4**. For the OCEBM criteria, seven studies qualified as level II (Sands et al., 2008; Rubini et al., 2011; de la Cruz-Torres et al., 2019; Balcı et al., 2020; Durukan et al., 2025; Jochum et al., 2025; Yan et al., 2025), and 16 studies qualified as level III (Di Cagno et al., 2010; Agopyan et al., 2012; Agopyan et al., 2013; Morrin and Redding, 2013; Williams et al., 2013; Cengiz et al., 2014; Donti et al., 2014; Silva et al., 2014; Johnson et al., 2018; Papia et al., 2018; Bogdanis et al., 2019; Dallas et al., 2019; Pessali-Marques et al., 2020; Arı, 2021; Dierick et al., 2021; Kurt et al., 2024). As most included studies were classified as Level III, the overall certainty of the evidence is limited and the findings should be interpreted cautiously.

### 3.3 Stretching characteristics

The included studies investigated a wide range of stretching techniques, encompassing static stretching (SS), dynamic stretching (DS), proprioceptive neuromuscular facilitation (PNF) stretching, neural sliding, intermittent and continuous stretching, passive stretching, constant torque stretching and combined stretching. All the included studies focused on single-session interventions, with an average duration of 7.3 ± 5.1 min (range: 0.7–22 min). The average net stretching time was 94.2 ± 72.1 s for SS, 83.3 ± 59.9 s for DS, and 90.0 ± 30.0 s for PNF protocols. Stretching protocols varied substantially in terms of repetition schemes, hold durations, intensity, and tempo. For SS, the common durations per repetition were 30 s and 90 s, often performed in sets of 2 × 30 s, 3 × 30 s, 4 × 30 s, or 6 × 30 s. DS was typically performed for durations of 10–40 s per bout and sometimes incorporated different tempos, including slow, moderate, or fast speeds. PNF stretching frequently followed contract-relax techniques, with repetitions such as 4 × 10-10–10 s and 2 × 30 s. Additional protocols examined included neural sliding, which was generally performed in sets of 3 × 60 s. Moreover, combined stretching methods were also explored for their potential synergistic effects, including alternating SS and DS within a single set (e.g., 15 s SS + 15 s DS, 30 s SS + 30 s DS). Stretching intensity was poorly reported across the included studies. Although a few studies described intensity using subjective terms such as “mild discomfort” or “tension but not pain,” the majority did not report or standardize the prescribed stretching intensity, precluding meaningful synthesis of this variable.

### 3.4 Acute effects of stretching on flexibility and performance

This systematic review provides a comprehensive examination of distinct outcomes across flexibility and performance parameters, drawing from a robust dataset of 23 included studies. The findings reveal a diverse array of results across the different measures, offering insights into the effectiveness of the various interventions. Specifically, flexibility showed significant improvements in 12 studies (Sands et al., 2008; Rubini et al., 2011; Williams et al., 2013; Donti et al., 2014; Papia et al., 2018; Bogdanis et al., 2019; de la Cruz-Torres et al., 2019; Balcı et al., 2020; Pessali-Marques et al., 2020; Dierick et al., 2021; Jochum et al., 2025; Yan et al., 2025), while two studies reported a significant decrease (Morrin and Redding, 2013; Johnson et al., 2018), and two others found no significant effects (Arı, 2021; Dierick et al., 2021). Four studies reported significant performance enhancements (Agopyan et al., 2013; Dallas et al., 2019; Arı, 2021; Durukan et al., 2025), whereas five studies indicated significant declines (Di Cagno et al., 2010; Agopyan et al., 2013; Cengiz et al., 2014; Silva et al., 2014; Bogdanis et al., 2019), and 12 studies observed no statistically meaningful impacts (Di Cagno et al., 2010; Agopyan et al., 2012; Agopyan et al., 2013; Morrin and Redding, 2013; Donti et al., 2014; Johnson et al., 2018; Papia et al., 2018; de la Cruz-Torres et al., 2019; Arı, 2021; Dierick et al., 2021; Kurt et al., 2024; Yan et al., 2025).

Moreover, three studies compared different stretching protocols with one another. Sands et al. (2008) found that adding vibration to SS (SS + vibration) significantly improved passive flexibility compared to SS alone (p = 0.002). Cengiz et al. (2014) compared SS and DS protocols and found that DS resulted in a significantly greater power deficit than SS (p < 0.05). Durukan et al. (2025) compared SS and DS protocols and found that dynamic stretching resulted in significantly better dynamic balance performance compared to static stretching (p = 0.023).

### 3.5 Moderating variables in stretching intervention studies on flexibility and performance

#### 3.5.1 Type of stretching

For SS interventions, a moderate mean effect of 2.97% (95% CI: 2.13–3.81%) was observed for flexibility from pre- to post-intervention (i.e., no control group), based on data synthesized from seven studies. Among these studies, six studies reported significant improvements (Rubini et al., 2011; Williams et al., 2013; Donti et al., 2014; Papia et al., 2018; Bogdanis et al., 2019; de la Cruz-Torres et al., 2019), while one study found a significant negative effect (Johnson et al., 2018), and another reported no significant effect (Papia et al., 2018). In addition, when comparing the intervention group to the control group, five studies demonstrated a moderate mean effect of 2.36% (95% CI: 0.73–3.98%). Among these studies, two showed significant improvements (Jochum et al., 2025; Yan et al., 2025), while the others reported a significant decline or no significant effects (Morrin and Redding, 2013; Arı, 2021; Dierick et al., 2021). Regarding performance from pre- to post-intervention, SS interventions showed a small mean effect of −0.88% (95% CI: −2.17 to 0.40%), based on data from six studies. Among these studies, only one study reported significant improvements (Agopyan et al., 2013), while two studies demonstrated significant negative effects (Agopyan et al., 2013; Bogdanis et al., 2019). In addition, five studies found no significant effects (Agopyan et al., 2013; Donti et al., 2014; Johnson et al., 2018; Papia et al., 2018; de la Cruz-Torres et al., 2019). In comparison to the control group, the intervention group showed a trivial mean effect of −0.07% (95% CI: −0.39 to 0.25%) across six studies. Among these studies, three studies showed significant negative effects (Di Cagno et al., 2010; Silva et al., 2014; Arı, 2021), and five studies found no significant effects (Di Cagno et al., 2010; Agopyan et al., 2012; Morrin and Redding, 2013; Arı, 2021; Yan et al., 2025). For flexibility, DS interventions showed a small mean effect of 0.77% (95% CI: −2.11% to 3.65%), compared to the control group, based on data from four studies. Among these studies, two demonstrated a significant positive effect (Dierick et al., 2021; Yan et al., 2025), one showed a significant negative effect (Morrin and Redding, 2013), and only one reported no significant effect (Arı, 2021). Regarding performance, the DS intervention group showed a small mean effect of 0.55% (95% CI: −1.10% to 2.20%), compared to the control group, based on data from four studies. One study reported a significant positive effect (Arı, 2021), while the other three demonstrated no significant effects (Morrin and Redding, 2013; Kurt et al., 2024; Yan et al., 2025). According to two studies, PNF interventions produced a small mean effect of 1.04% (95% CI: −0.52% to 2.61%) on performance when compared with control conditions. One study demonstrated a significant negative effect (Silva et al., 2014), while the other showed no significant effect (Yan et al., 2025). For combined stretching interventions, a small mean effect of 1.65% (95% CI: −1.18% to 4.47%) on flexibility was observed, compared to the control group, based on data from two studies. Both studies reported no significant effects (Morrin and Redding, 2013; Arı, 2021). Regarding performance, the intervention group showed a small mean effect of 0.60% (95% CI: −0.33% to 1.53%), based on pooled data from two studies. One study demonstrated a significant positive effect (Arı, 2021), while another found no significant effect (Morrin and Redding, 2013).

#### 3.5.2 Stretching duration

The effect of stretching duration varied, depending on whether stretches were held for less than or equal to 60 s or more than 60 s:

Short-duration stretches (≤60 s): For performance, the interventions yielded a trivial mean effect of 0.06% (95% CI: −0.01 to 0.13), analyzed across two studies. While one study reported a significant positive effect, the other two ones found no significant impact (Donti et al., 2014; Dallas et al., 2019). The interventions demonstrated a small mean effect of 0.79% (95% CI: −0.39 to 1.97%) on flexibility, compared to the control group, based on data from four studies. Among these studies, one study reported a significant positive effect (Williams et al., 2013), while the other showed significant negative effect (Morrin and Redding, 2013). Moreover, three studies revealed no significant effects (Morrin and Redding, 2013; Arı, 2021; Dierick et al., 2021). Regarding performance, the intervention group showed a trivial mean effect of 0.48% (95% CI: −0.03 to 0.99), synthesized from four studies. Among these studies, one study reported a significant positive effect (Arı, 2021), two studies found significant negative effects (Silva et al., 2014; Arı, 2021), and three studies showed no significant effects (Agopyan et al., 2012; Morrin and Redding, 2013; Arı, 2021).

Long-duration stretches (>60 s): For flexibility, the interventions demonstrated a large mean effect of 6.21% (95% CI: 2.68% to 9.75%), synthesized from six studies comparing pre- and post-intervention results. Among these studies, five studies showed significant positive effects (Rubini et al., 2011; Papia et al., 2018; Bogdanis et al., 2019; de la Cruz-Torres et al., 2019; Balcı et al., 2020), while one study found a significant negative effect (Johnson et al., 2018), and another showed no significant effect (Papia et al., 2018). For performance, the interventions yielded a small mean effect of −1.80% (95% CI: −3.97% to 0.37%), aggregated from five studies. Among these studies, one study showed a significant positive effect (Agopyan et al., 2013), two studies demonstrated significant negative effects (Agopyan et al., 2013; Bogdanis et al., 2019), and four studies showed no significant effects (Agopyan et al., 2013; Johnson et al., 2018; Papia et al., 2018; de la Cruz-Torres et al., 2019). The interventions demonstrated a small mean improvement of 1.89% (95% CI: 0.01% to 3.78%) in flexibility, compared to the control group, based on data from two studies. All of them reported significant positive effects (Jochum et al., 2025; Yan et al., 2025). Regarding performance, the intervention group showed a trivial mean effect of −0.02% (95% CI: −0.03 to -0.01), based on data from two studies. One reported significant negative effects (Di Cagno et al., 2010), and another one showed no significant effects (Yan et al., 2025).

## 4 Discussion

This systematic review investigated the acute effects of stretching type and duration on flexibility and performance in athletes with high ROM demands. The synthesized results from 23 included studies indicated that SS, when assessed from pre- to post-intervention measurements and compared to a control group, produced moderate improvements in flexibility. However, performance outcomes following SS demonstrated a small negative effect. Compared to control conditions, SS had a trivial negative overall effect on performance. In contrast, DS showed small effects on flexibility compared to control, and yielded small positive effects on performance. Combined stretching protocols demonstrated small effects on both flexibility and performance. PNF interventions produced a small positive effect on performance compared to control conditions. When evaluating stretching duration, a clearer pattern emerged. Short-duration stretches (≤60 s) resulted in a small flexibility improvement when compared to a control group, and trivial improvements in performance from pre- to post-intervention and compared to control. Long-duration stretches (>60 s) were associated with large improvements in flexibility from pre- to post-intervention and a small improvement compared to control, but also showed small declines in performance from pre- to post-intervention and trivial effects relative to control. These findings suggest that while both stretching type and duration can affect flexibility, performance effects are more variable and often depend on the stretch duration and the context in which it is applied.

### 4.1 Type of stretching: differential effects on flexibility and performance

This systematic review revealed distinct and measurable differences in how the various types of stretching affect flexibility and performance in athletes with high ROM demands. Among the interventions, SS demonstrated the greatest improvements in flexibility, with moderate changes. These findings align with prior studies, including Behm et al. (2016), Bryant et al. (2023) and Ingram et al. (2025b), which have consistently shown that SS enhances flexibility through mechanisms such as increased stretch tolerance, reduced passive resistance in musculotendinous units, and viscoelastic changes in connective tissue. Weppler and Magnusson (2010) further noted that sustained muscle elongation can acutely increase ROM by altering stretch perception and muscle stiffness. However, these findings appear to diverge from research on the general population. For example, Behm et al. (2023) found that both SS and DS produced comparable increases in ROM in untrained and recreationally active individuals. In contrast, the current review found that only SS led to meaningful flexibility improvements in athletes with high ROM demands, while DS produced only small gains. This discrepancy can be attributed to population-specific factors such as training background, baseline flexibility, neuromuscular conditioning, or potentially to the limited number of DS-focused studies included in this review. As such, general population outcomes should be cautiously interpreted when applied to flexibility-trained athletes.

Despite these flexibility benefits, SS exhibited trivial to small but consistent negative impacts on athletic performance. These performance decrements, which were particularly observed in vertical jump height and isokinetic strength, are likely due to neuromuscular inhibition, including reduced muscle activation, decreased motor unit firing frequency, and diminished muscle-tendon unit stiffness. Studies such as Trajano et al. (2014) and Simic et al. (2013) have demonstrated that these neural and mechanical changes are particularly pronounced after long-duration SS, impairing the efficiency of force transmission and explosive movement execution.

DS, in contrast, was associated with small flexibility gains and small performance improvements. These performance benefits were most evident in sprinting and vertical jump tasks (Dallas et al., 2019; Arı, 2021). The favorable effects of DS can be attributed to enhanced muscle temperature, increased nerve conduction velocity, elevated enzymatic activity, and post-activation potentiation (Hodgson et al., 2005; Blazevich and Babault, 2019), which collectively improve muscle contractility and reduce electromechanical delay (Blazevich and Babault, 2019). Furthermore, DS often replicates sport-specific movement patterns, facilitating neuromuscular coordination and proprioceptive readiness. This is supported by findings from Dallas et al. (2019), who observed improved short-distance sprint times following DS protocols (*d* = 0.26 and 0.09).

The differential impact of SS and DS reinforces the need to match the type of stretching to the athlete’s immediate goal. For enhancing flexibility—especially in sports such as gymnastics, ballet, and martial arts—SS remains a valuable tool. However, if the goal is to optimize performance in power-based activities, DS is more appropriate, due to its priming effects on the neuromuscular system. Notably, the potential negative impact of SS on performance can be mitigated by following it with DS or sport-specific dynamic drills, as suggested by Behm et al. (2016), who advocated this approach to restore muscle activation and dynamic readiness (Behm et al., 2016).

Combined stretching protocols, such as alternating SS and DS, showed only small benefits for both flexibility and performance. While these findings suggest a potentially synergistic effect, the results varied, depending on the sequencing and specific application. For instance, it has been found that SS followed by dynamic movement can preserve flexibility gains while minimizing performance loss (Simic et al., 2013). However, more research is needed to establish optimal combinations and durations. PNF stretching produced a small positive effect on performance compared to control conditions. This suggests that PNF may be a viable alternative for athletes seeking performance enhancement without the performance decrements associated with prolonged SS. Although all included athletes required high ROM, the included sports differed substantially in their performance demands. The reviewed studies involved athletes from aesthetic and technical sports (e.g., gymnastics, ballet, and dance), combat sports (e.g., wrestling), and aquatic sports (e.g., swimming). Consequently, the relative importance of flexibility, strength, power, balance, and technical execution likely varied across studies and may have contributed to the heterogeneous findings. Therefore, the results should be interpreted as general recommendations for high-ROM athletes rather than sport-specific prescriptions.

In summary, the type of stretching exerts a significant and differentiated effect on both flexibility and performance. SS is effective in improving ROM but can temporarily impair strength and power. DS was associated with small performance improvements and limited flexibility gains. Although these findings suggest that DS may be beneficial in pre-competition routines, the available evidence in athletes with high ROM demands remains limited and further research is required. The evidence regarding PNF remains limited. While a small positive mean effect was observed, this finding was based on a small number of studies with mixed results; therefore, no firm conclusions can be drawn regarding its effects on performance. Combined approaches can offer balanced benefits, depending on their design. Practitioners should tailor stretching protocols based on sport-specific demands, session goals (e.g., warm-up vs. recovery), and individual athlete characteristics, to optimize outcomes.

### 4.2 Stretching duration: differential effects on flexibility and performance

The findings of this review demonstrated a clear duration-dependent effect of stretching on flexibility and performance in athletes with high ROM demands. Specifically, long-duration stretching interventions (>60 s) were consistently effective in improving flexibility outcomes. The analysis revealed a large mean effect from pre- to post-intervention; however, when compared to control conditions, the effect was small. These results underscore the effectiveness of prolonged stretching in acutely increasing joint ROM and muscular extensibility. These findings align with previous evidence from Simpson et al. (2017), who showed that a long stretching duration (180 s) produced significant increases in ROM for the ankle joint (14.94%, *d* = 2.05)]. Similarly, Behm et al. (2016) and Simic et al. (2013) found that long stretching durations acutely reduced strength and power output, particularly in sprint and jump performance. This review mirrors these observations, showing a small negative mean effect on performance, with some studies (>60 s) reporting significant decrements in isokinetic strength and vertical jump (Agopyan et al., 2013; Bogdanis et al., 2019). These effects are likely attributable to decreased muscle-tendon stiffness, reduced motor unit recruitment, and transient neural inhibition following prolonged muscle elongation (Weppler and Magnusson, 2010; Trajano et al., 2014; Behm et al., 2016).

In contrast, short-duration SS (≤60 s) demonstrated small flexibility improvements, with trivial to small performance effects. However, the results were mixed. For instance, Donti et al. (2014) reported flexibility benefits in gymnasts after short-duration stretches, without compromising countermovement jump performance (*p* > 0.05) (Donti et al., 2014). Conversely, Silva et al. (2014) found that even brief stretching reduced swimming performance in competitive swimmers (SS: *d* = 0.29; PNF: *d* = 0.46) (Silva et al., 2014). These discrepancies suggest that the specific muscle groups stretched, the sport context, and athlete characteristics can influence the extent of performance impairments, even when using short-duration protocols.

Moreover, several studies (e.g., Behm et al., 2016) have suggested that incorporating dynamic activities following SS can help mitigate potential impairments in strength and power, particularly when SS is included in a warm-up routine (Behm et al., 2016). This highlights the importance of sequencing within warm-up design, where short-duration SS can be incorporated as long as it is followed by dynamic drills or sport-specific movements to restore neuromuscular readiness.

From a practical standpoint, for athletes aiming to enhance flexibility, long-duration stretching (>60 s) should be incorporated into separate sessions or off-day training (Konrad et al., 2024). Sports such as ballet, swimming, wrestling, and gymnastics—where ROM is a key factor—can greatly benefit from prolonged stretching, even if there is, acutely, a minor trade-off in performance. On the other hand, short-duration stretching (≤60 s) is best used pre-competition. It poses less risk to performance and can be incorporated into warm-up routines, particularly when paired with DS, to activate muscles and prepare for explosive efforts (Behm et al., 2016). The most important takeaway is that high-ROM athletes need tailored protocols that reflect the demands of their sport. While this review emphasizes the unique needs of high-ROM athletes, the insights gained can also inform training approaches for the general population. Traditional stretching protocols often adopt a one-size-fits-all approach, but the findings from this review highlight the importance of sport-specific adjustments. The findings suggest that athletes with high ROM demands may respond differently to stretching than the general population, highlighting the need for sport-specific recommendations. For example, the stretching strategies that work for a gymnast may not be the best for a swimmer, and understanding these distinctions is key to optimizing both flexibility and performance.

### 4.3 Limitations

Several limitations should be acknowledged. Most notably, the methodological quality of the included studies was limited: 15 out of 23 studies were rated as low quality, while only four studies were rated as high quality. Common issues included poor reporting on participant selection processes, inadequate blinding, and small sample sizes, which can introduce bias and reduce the generalizability of the results. Furthermore, the OCEBM assessment showed that only seven studies were classified as Level II evidence, whereas the majority were classified as Level III. This suggests that the current evidence base is composed predominantly of studies with a greater susceptibility to bias and confounding than higher-level evidence. Therefore, while the overall findings consistently support the beneficial effects of stretching on flexibility and the differential effects of stretching modalities on performance, the certainty of these conclusions should be considered moderate rather than high. Additional well-designed RCTs are needed to strengthen confidence in these findings and improve the overall level of evidence. In addition, considerable heterogeneity existed across the studies in terms of stretching protocols (e.g., intensity, tempo, number of repetitions), outcome measures, and sport-specific demands, which complicates direct comparisons and limits the precision of pooled estimates. Moreover, although all included athletes participated in sports requiring high ROM, substantial heterogeneity existed across sport categories (e.g., aesthetic/technical, combat, and aquatic sports) and even within sports like track and field, where our classification was deliberately restricted to pole vault and high jump to maintain focus on events with the strongest biomechanical evidence for ROM demands; extending it to include events like hurdling would have introduced excessive heterogeneity. The sport-specific search strategy may have limited the identification of relevant studies in athletes from sports not explicitly included in the search terms. However, supplementary search methods were employed to mitigate this risk. Due to the limited number of studies within each category, formal sport-specific analyses were not feasible. Another limitation is the exclusive focus on acute effects, which precludes conclusions about the long-term implications of stretching interventions on chronic flexibility adaptations or performance trends. Furthermore, due to substantial heterogeneity in the specific muscle groups stretched across studies, we were unable to include this variable as a moderator in our analysis, nor did we account for other moderating variables such as athlete training level, baseline flexibility, or sport-specific warm-up routines following stretching, which may have influenced performance outcomes. Another important limitation was the inconsistent reporting of stretching intensity. Although a few studies described stretching intensity using subjective terms, the majority did not report or standardize this key intervention parameter. Consequently, stretching intensity could not be examined as a potential moderating variable or its contribution to the observed flexibility and performance outcomes. This lack of standardized reporting limits the interpretation of potential dose–response relationships and reduces the reproducibility and comparability of findings across studies. Recent methodological commentaries have highlighted inadequate reporting of stretching intensity as a persistent limitation in stretching research and have advocated for standardized methods of prescribing and reporting this important training variable (Ingram et al., 2025a; Warneke et al., 2025). Future studies should therefore clearly define and quantify stretching intensity using reproducible approaches to improve the quality, reproducibility, and interpretability of the evidence. Also, the inclusion of uncontrolled pre-post studies, which by design lack a control group, increases the risk of confounding and limits the ability to attribute observed changes solely to the stretching intervention, thereby reducing the overall certainty of the evidence. This should be considered when interpreting the findings. Lastly, the limited number of studies examining dynamic, combined, or PNF stretching modalities restricts our ability to draw strong conclusions about these approaches, particularly regarding their performance implications. In summary, this review provides a practical synthesis of the acute effects of the various stretching protocols in high-ROM athletes. However, the conclusions should be interpreted cautiously due to study heterogeneity, potential biases, limited high-quality evidence, and the inconsistent reporting of key intervention variables, particularly stretching intensity. Future research should prioritize well-designed RCTs with standardized stretching protocols and comprehensive reporting of intervention characteristics, including stretching intensity, to strengthen causal inferences. Studies should employ sport-specific performance tests that are relevant to the athletes’ competitive demands and should assess both acute and chronic outcomes to determine the sustainability of flexibility and performance effects. Additionally, larger sample sizes and improved reporting of participant characteristics and intervention details are needed to enhance generalizability, reproducibility, and comparability across studies.

## 5 Conclusion

This systematic review underscores the nuanced effects of acute stretching interventions in athletes who require high flexibility. SS improved ROM but may negatively affect performance, Conversely, DS may offer small flexibility benefits with favorable performance effects, suggesting it may be more suitable for pre-competition warm-ups. However, given that the majority of included studies were rated as low quality (15 studies), these findings should be interpreted cautiously, and stronger recommendations await confirmation from well-designed RCTs. The findings also highlight the importance of tailoring stretching strategies based on sport-specific demands, session goals, and performance priorities. Coaches and practitioners should carefully consider both the type and duration of stretching to strike an optimal balance between flexibility enhancement and performance maintenance. Future research should further explore combined protocols and their application across different athletic populations, with greater emphasis on standardization and longitudinal outcomes.

## Data Availability

The original contributions presented in the study are included in the article/**Supplementary Material**. Further inquiries can be directed to the corresponding author.

## References

[B1] Afonso J. Andrade R. Rocha-Rodrigues S. Nakamura F. Y. Sarmento H. Freitas S. R. . (2024). What we do not know about stretching in healthy athletes: a scoping review with evidence gap map from 300 trials. Sports Med. 54, 1517–1551. doi: 10.1007/s40279-024-02002-7 38457105 PMC11239752

[B2] Agopyan A. Bozdogan F. S. Tekin D. Yetgin M. K. Guler C. G. (2012). Acute effects of static stretching exercises on short-distance flutter kicking time in child swimmers. Int. J. Perform. Anal. Sport 12, 484–497. doi: 10.1080/24748668.2012.11868613 37339054

[B3] Agopyan A. Tekin D. Ünal M. Kurtel H. Turan G. Ersöz A. (2013). Acute effects of static stretching on isokinetic thigh strenght on modern dancers. J. Sports Med. Phys. Fitness 53, 538–550. 23903535

[B4] Alzahrani H. Mackey M. Stamatakis E. Zadro J. R. Shirley D. (2019). The association between physical activity and low back pain: a systematic review and meta-analysis of observational studies. Sci. Rep. 9, 8244. doi: 10.1038/s41598-019-44664-8 31160632 PMC6547713

[B5] Arı Y. (2021). Effects of different stretching methods on speed, jump, flexibility and upper extremity performance in wrestlers. Kinesiologia Slovenica. 27, 162–176. doi: 10.2204/iodp.proc.304305.102.2006 41596867

[B6] Babault N. Rodot G. Champelovier M. Cometti C. (2021). A survey on stretching practices in women and men from various sports or physical activity programs. Int. J. Environ. Res. Public Health 18, 3928. doi: 10.3390/ijerph18083928 33918033 PMC8068839

[B7] Balcı A. Ünüvar E. Akınoğlu B. Kocahan T. (2020). The effect of different neural mobilization exercises on hamstring flexibility and functional flexibility in wrestlers. J. Exerc Rehabil. 16, 503. doi: 10.12965/jer.2040700.350 33457386 PMC7788253

[B8] Behm D. G. Alizadeh S. Daneshjoo A. Anvar S. H. Graham A. Zahiri A. . (2023). Acute effects of various stretching techniques on range of motion: A systematic review with meta-analysis. Sports Med. - Open 9, 107. doi: 10.1186/s40798-023-00652-x 37962709 PMC10645614

[B9] Behm D. G. Blazevich A. J. Kay A. D. McHugh M. (2016). Acute effects of muscle stretching on physical performance, range of motion, and injury incidence in healthy active individuals: a systematic review. Appl. Physiol. Nutr. Metab. 41, 1–11. doi: 10.1139/apnm-2015-0235 26642915

[B10] Blazevich A. J. Babault N. (2019). Post-activation potentiation versus post-activation performance enhancement in humans: historical perspective, underlying mechanisms, and current issues. Front. Physiol. 10, 1359. doi: 10.3389/fphys.2019.01359 31736781 PMC6838751

[B11] Blazevich A. J. Gill N. D. Kvorning T. Kay A. D. Goh A. M. Hilton B. . (2018). No effect of muscle stretching within a full, dynamic warm-up on athletic performance. Med. Sci. Sports Exerc. 50, 1258–1266. doi: 10.1249/mss.0000000000001539 29300214

[B12] Bogdanis G. C. Donti O. Tsolakis C. Smilios I. Bishop D. J. (2019). Intermittent but not continuous static stretching improves subsequent vertical jump performance in flexibility-trained athletes. J. Strength Cond Res. 33, 203–210. doi: 10.1519/jsc.0000000000001870 28240710

[B13] Bryant J. Cooper D. J. Peters D. M. Cook M. D. (2023). The effects of static stretching intensity on range of motion and strength: A systematic review. J. Funct. Morphol. Kinesiol 8, 37. doi: 10.3390/jfmk8020037 37092369 PMC10123604

[B14] Cengiz A. Demirhan B. Yamaner F. Kir R. (2014). Acute effects of dynamic versus static stretching on aneorobic power and muscle damage of wrestlers. Anthropologist 18, 885–891. doi: 10.1080/09720073.2014.11891620 37339054

[B15] Chaabene H. Behm D. G. Negra Y. Granacher U. (2019). Acute effects of static stretching on muscle strength and power: an attempt to clarify previous caveats. Front. Physiol. 10, 489981. doi: 10.3389/fphys.2019.01468 31849713 PMC6895680

[B16] Chen S.-M. Liu M.-F. Cook J. Bass S. Lo S. K. (2009). Sedentary lifestyle as a risk factor for low back pain: a systematic review. Int. Arch. Occup. Environ. Health 82, 797–806. doi: 10.1007/s00420-009-0410-0 19301029

[B17] Cohen J. (1968). Weighted kappa: nominal scale agreement provision for scaled disagreement or partial credit. Psychol. Bull. 70, 213. doi: 10.1037/h0026256 19673146

[B18] Dallas G. Theodorou A. Paradisis G. (2019). The effect of different duration of dynamic stretching on sprint run and agility test on female gymnast. J. Phys. Educ. Sport. 19, 268–272.

[B19] Deeks J. J. Dinnes J. D'Amico R. Sowden A. J. Sakarovitch C. Song F. . (2003). Evaluating non-randomised intervention studies. Health Technol. Assess. (Winchester England). 7, iii–173. doi: 10.3310/hta7270 14499048

[B20] de la Cruz-Torres B. Barrera-García-Martín I. Albornoz-Cabello M. (2019). Immediate effects of ultrasound-guided percutaneous neuromodulation versus physical exercise on performance of the flexor hallucis longus muscle in professional dancers: a randomised clinical trial. Acupunct Med. 37, 91–97. doi: 10.1177/0964528419826103 30860393

[B21] Di Cagno A. Baldari C. Battaglia C. Gallotta M. C. Videira M. Piazza M. . (2010). Preexercise static stretching effect on leaping performance in elite rhythmic gymnasts. J. Strength Cond Res. 24, 1995–2000. doi: 10.1519/jsc.0b013e3181e34811 20634743

[B22] Dierick F. Buisseret F. Filiputti L. Roussel N. (2021). Kinematics and esthetics of grand battement after static and dynamic hamstrings stretching in adolescents. Motor Control. 25, 403–422. doi: 10.1123/mc.2020-0101 33837160

[B23] Donti O. Tsolakis C. Bogdanis G. C. (2014). Effects of baseline levels of flexibility and vertical jump ability on performance following different volumes of static stretching and potentiating exercises in elite gymnasts. J. Sports Sci. Med. 13, 105. 24570613 PMC3918545

[B24] Downs S. H. Black N. (1998). The feasibility of creating a checklist for the assessment of the methodological quality both of randomised and non-randomised studies of health care interventions. J. Epidemiol. Community Health 52, 377–384. doi: 10.1136/jech.52.6.377 9764259 PMC1756728

[B25] Durukan E. Aydin G. Goktepe M. Cicek G. Guder F. Isik O. (2025). Comparison of the acute effects of static and dynamic stretching exercises on the balance performance of Turkish wrestlers. BMC Sports Sci. Med. Rehab. 17, 242. doi: 10.1186/s13102-025-01293-1 40818950 PMC12357431

[B26] Franchini E. Herrera-Valenzuela T. (2021). Developing flexibility for combat sports athletes. Rev. Artes Marciales Asiáticas. 16, 192–203. doi: 10.18002/rama.v16i1s.7005

[B27] Haddad M. Abbes Z. Mujika I. Chamari K. (2021). Impact of COVID-19 on swimming training: practical recommendations during home confinement/isolation. Int. J. Environ. Res. Public Health 18, 4767. doi: 10.3390/ijerph18094767 33947100 PMC8124287

[B28] Halder K. Chatterjee A. Pal R. Tomer O. S. Saha M. (2015). Age related differences of selected Hatha yoga practices on anthropometric characteristics, muscular strength and flexibility of healthy individuals. Int. J. Yoga. 8, 37–46. doi: 10.4103/0973-6131.146057 25558132 PMC4278134

[B29] Hodgson M. Docherty D. Robbins D. (2005). Post-activation potentiation: underlying physiology and implications for motor performance. Sports Med. 35, 585–595. doi: 10.2165/00007256-200535070-00004 16026172

[B30] Howick J. Chalmers I. Glasziou P. (2011). OCEBM levels of evidence working group ‘The oxford 2011 levels of evidence’: oxford centre for evidence-based medicine. Univ. Oxford: Oxford UK.

[B31] Ingram L. A. Tomkinson G. R. d’Unienville N. M. A. Gower B. Gleadhill S. Boyle T. . (2025a). Optimising the dose of static stretching to improve flexibility: A systematic review, meta-analysis and multivariate meta-regression. Sports Med. 55, 597–617. doi: 10.1007/s40279-024-02143-9 39614059

[B32] Ingram L. A. Tomkinson G. R. d’Unienville N. M. Gower B. Gleadhill S. Boyle T. . (2025b). Mechanisms underlying range of motion improvements following acute and chronic static stretching: A systematic review, meta-analysis and multivariate meta-regression. Sports Med., 1–18. doi: 10.1007/s40279-025-02204-7 40180774 PMC12152101

[B33] Iranmanesh M. Hosseini E. Bigtashkhani R. Sabouri A. Alghosi M. Alimoradi M. . (2025). The role of stretching protocols in post-fatigue performance and flexibility among soccer players. Sci. Rep. 16, 2499. doi: 10.1038/s41598-025-32188-3 41419757 PMC12819411

[B34] Jochum D. Vogel V. Warneke K. (2025). Acute effects of passive stretching with and without vibration on hip range of motion, temperature, and stiffness parameters in male elite athletes. J. Funct. Morphol. Kinesiol. 10, 17. doi: 10.3390/jfmk10010017 39846658 PMC11755640

[B35] Johnson A. W. Warcup C. N. Seeley M. K. Eggett D. Feland J. B. (2018). The acute effects of stretching with vibration on dynamic flexibility in young female gymnasts. J. Sports Med. Phys. Fitness 59, 210–216. doi: 10.23736/s0022-4707.18.08290-7 29327828

[B36] Kapo S. Smajlović N. Kajmović H. Ćirić A. Ćutuk M. Bejdić A. (2016). Effects of different stretching protocols on knee muscles strength and power parameters measured by Biodex dynamometer. Tehnički Vjesnik. 23, 273–278. doi: 10.17559/TV-20150506151811

[B37] Kay A. D. Blazevich A. J. (2012). Effect of acute static stretch on maximal muscle performance: a systematic review. Med. Sci. Sports Exerc. 44, 154–164. doi: 10.1249/mss.0b013e318225cb27 21659901

[B38] Konrad A. Alizadeh S. Daneshjoo A. Anvar S. H. Graham A. Zahiri A. . (2024). Chronic effects of stretching on range of motion with consideration of potential moderating variables: A systematic review with meta-analysis. J. Sport Health Sci. 13, 186–194. doi: 10.1016/j.jshs.2023.06.002 37301370 PMC10980866

[B39] Kurt C. Tuna G. Kurtdere İ. (2024). Acute effects of slow, moderate and fast tempo dynamic stretching exercises on power in well-trained male wrestlers. J. Hum. Kinet. 93, 155. doi: 10.5114/jhk/183543 39132429 PMC11307189

[B40] Lima C. D. Ruas C. V. Behm D. G. Brown L. E. (2019). Acute effects of stretching on flexibility and performance: a narrative review. J. Sci. Sport Exerc. 1, 29–37. doi: 10.1007/s42978-019-0011-x 30311153

[B41] McNeal J. R. Sands W. A. (2006). Stretching for performance enhancement. Curr. Sports Med. Rep. 5, 141–146. doi: 10.1097/01.csmr.0000306304.25944.07 16640950

[B42] Methley A. M. Campbell S. Chew-Graham C. McNally R. Cheraghi-Sohi S. (2014). PICO, PICOS and SPIDER: a comparison study of specificity and sensitivity in three search tools for qualitative systematic reviews. BMC Health Serv. Res. 14, 1–10. doi: 10.1186/s12913-014-0579-0 25413154 PMC4310146

[B43] Morrin N. Redding E. (2013). Acute effects of warm-up stretch protocols on balance, vertical jump height, and range of motion in dancers. J. Dance Med. Sci. 17, 34–40. doi: 10.12678/1089-313x.17.1.34 23498355

[B44] Munn J. Sullivan S. J. Schneiders A. G. (2010). Evidence of sensorimotor deficits in functional ankle instability: a systematic review with meta-analysis. J. Sci. Med. Sport. 13, 2–12. doi: 10.1016/j.jsams.2009.03.004 19442581

[B45] Page M. J. McKenzie J. E. Bossuyt P. M. Boutron I. Hoffmann T. C. Mulrow C. D. . (2021). The PRISMA 2020 statement: an updated guideline for reporting systematic reviews. Int. J. Surg. 88, 105906. doi: 10.31222/osf.io/v7gm2_v1 33789826

[B46] Papia K. Bogdanis G. C. Toubekis A. Donti A. Donti O. (2018). Acute effects of prolonged static stretching on jumping performance and range of motion in young female gymnasts. Sci. Gym J. 10, 217–226. doi: 10.52165/sgj.10.2.217-226

[B47] Pessali-Marques B. Peixoto G. H. Cabido C. E. André Gustavo P. A. Rodrigues S. A. Tourino F. D. . (2020). Biomechanical response to acute stretching in dancers and non-dancers. J. Dance Med. Sci. 24, 12–18. doi: 10.12678/1089-313x.24.1.12 32093820

[B48] Rubini E. C. Souza A. C. Mello M. L. Bacurau R. F. Cabrai L. R. Farinatti P. T. (2011). Immediate effect of static and proprioceptive neuromuscular facilitation stretching on hip adductor flexibility in female ballet dancers. J. Dance Med. Sci. 15, 177–181. doi: 10.1177/1089313x1101500406 22687658

[B49] Rudisill S. S. Varady N. H. Kucharik M. P. Eberlin C. T. Martin S. D. (2023). Evidence-based hamstring injury prevention and risk factor management: a systematic review and meta-analysis of randomized controlled trials. Am. J. Sports Med. 51, 1927–1942. doi: 10.1177/03635465221083998 35384731

[B50] Ruggieri R. M. Costa P. B. (2019). Contralateral muscle imbalances and physiological profile of recreational aerial athletes. J. Funct. Morphol. Kinesiol. 4, 49. doi: 10.3390/jfmk4030049 33467364 PMC7739357

[B51] Sands W. A. McNeal J. R. Penitente G. Murray S. R. Nassar L. Jemni M. . (2016). Stretching the spines of gymnasts: a review. Sports Med. 46, 315–327. doi: 10.1007/s40279-015-0424-6 26581832 PMC4769315

[B52] Sands W. A. McNeal J. R. Stone M. H. Kimmel W. L. Gregory Haff G. Jemni M. (2008). The effect of vibration on active and passive range of motion in elite female synchronized swimmers. Eur. J. Sport Sci. 8, 217–223. doi: 10.1080/17461390802116682

[B53] Shrier I. (2005). When and whom to stretch? Gauging the benefits and drawbacks for individual patients. Phys. Sportsmedicine. 33, 22–26. doi: 10.3810/psm.2005.03.61 20086352

[B54] Silva G. C. E. Silveira A. Novaes J. Di Masi F. Conceição M. Dantas E. (2014). Acute effects of static and proprioceptive neuromuscular facilitation stretching on sprint performance in male swimmers. Med. Sport 67, 119–128.

[B55] Simic L. Sarabon N. Markovic G. (2013). Does pre‐exercise static stretching inhibit maximal muscular performance? A meta‐analytical review. Scand. J. Med. Sci. Sports 23, 131–148. doi: 10.1111/j.1600-0838.2012.01444.x 22316148

[B56] Simpson C. Kim B. Bourcet M. Jones G. Jakobi J. (2017). Stretch training induces unequal adaptation in muscle fascicles and thickness in medial and lateral gastrocnemii. Scand. J. Med. Sci. Sports 27, 1597–1604. doi: 10.1111/sms.12822 28138986

[B57] Stathokostas L. Little R. M. Vandervoort A. Paterson D. H. (2012). Flexibility training and functional ability in older adults: a systematic review. J. Aging Res. 2012, 306818. doi: 10.1155/2012/306818 23209904 PMC3503322

[B58] Trajano G. S. Seitz L. B. Nosaka K. Blazevich A. J. (2014). Can passive stretch inhibit motoneuron facilitation in the human plantar flexors? J. Appl. Physiol. 117, 1486–1492. doi: 10.1152/japplphysiol.00809.2014 25342705

[B59] Warneke K. Blazevich A. J. Jochum D. Behm D. G. Tomas E. Nakamura M. . (2025). Perception-based methods and beyond: A current opinion on how to assess static stretching intensity. Sports Med. 55, 2977–2986. doi: 10.1007/s40279-025-02307-1 40952622 PMC12628444

[B60] Warneke K. Lohmann L. H. (2024). Revisiting the stretch-induced force deficit: a systematic review with multilevel meta-analysis of acute effects: revisiting the stretch-induced force deficit. J. Sport Health Sci. 13 (6), 805–819. doi: 10.1016/j.jshs.2024.05.002 38735533 PMC11336295

[B61] Warneke K. Wirth K. Keiner M. Schiemann S. (2023). Improvements in flexibility depend on stretching duration. Int. J. Exerc Sci. 16, 83. doi: 10.70252/lbou2008 37113511 PMC10124737

[B62] Weppler C. H. Magnusson S. P. (2010). Increasing muscle extensibility: a matter of increasing length or modifying sensation? Phys. Ther. 90, 438–449. doi: 10.2522/ptj.20090012 20075147

[B63] Williams J. G. Laudner K. G. McLoda T. (2013). The acute effects of two passive stretch maneuvers on pectoralis minor length and scapular kinematics among collegiate swimmers. Int. J. Sports Phys. Ther. 8, 25. doi: 10.1249/01.mss.0000401380.54018.b3 23439770 PMC3578431

[B64] Yan R. Lin G. Peng W. Chen Y. Sun P. Sun J. . (2025). Time-dependent effects of acute stretching on power, balance, and flexibility in contemporary dancers: a randomized crossover trial. Sci. Rep. 15, 15489. doi: 10.1038/s41598-025-00027-0 40319037 PMC12049479

[B65] Zadro J. Shirley D. Ferreira M. Carvalho-Silva A. Lamb S. Cooper C. . (2017). Mapping the association between vitamin D and low back pain: a systematic review and meta-analysis of observational studies. Pain Physician. 20. doi: 10.36076/ppj/2017.7.611 29149142

