# Supplementary material for: How long should athletes with high range of motion demands stretch? Acute stretching durations for flexibility and performance: a systematic review
Source: Front Physiol. 2026 Jul 17;17:1881773. doi: 10.3389/fphys.2026.1881773 (PMC13423734; doi:10.3389/fphys.2026.1881773)
Supplement: Supplementary file 1 [file Table1.docx]

**Supplementary Table 1 : Search strings**

**Search string:**

(stretch*) AND (acute OR single OR immediate* OR sudden) AND (Gymanstic* OR Swim* OR Diving* OR "material art" OR Skat* OR SKiing* OR Danc* OR Climb* OR Wrestl* OR cheerlead* OR "Track and Field")

| **Databases** | **Search query** | **Number of paper** |
| --- | --- | --- |
| **Web of Science** | stretch* (Topic) AND acute OR single OR immediate* OR sudden (Topic) AND Gymanstic* OR Swim* OR Diving* OR "material art" OR Skat* OR SKiing* OR Danc* OR Climb* OR Wrestl* OR cheerlead* OR "Track and Field" (Topic) | **209** |
| **PubMed** | ((stretch*[Title/Abstract]) AND (acute[Title/Abstract] OR single[Title/Abstract] OR immediate*[Title/Abstract] OR sudden[Title/Abstract])) AND (Gymanstic*[Title/Abstract] OR Swim*[Title/Abstract] OR Diving*[Title/Abstract] OR "material art"[Title/Abstract] OR Skat*[Title/Abstract] OR SKiing*[Title/Abstract] OR Danc*[Title/Abstract] OR Climb*[Title/Abstract] OR Wrestl*[Title/Abstract] OR cheerlead*[Title/Abstract] OR "Track and Field"[Title/Abstract]) | **122** |
| **Scopus** | ( TITLE-ABS-KEY ( stretch* ) AND TITLE-ABS-KEY ( acute OR single OR immediate* OR sudden ) AND TITLE-ABS-KEY ( gymanstic* OR swim* OR diving* OR "material art" OR skat* OR skiing* OR danc* OR climb* OR wrestl* OR cheerlead* OR "Track and Field" ) ) | **293** |

**Supplementary Table 2: Excluded studies**

| **Population** | **Exclusion reason** |
| --- | --- |
| De Weijer, V. C., Gorniak, G. C., & Shamus, E. (2003). The effect of static stretch and warm-up exercise on hamstring length over the course of 24 hours. *Journal of Orthopaedic & Sports Physical Therapy*, *33*(12), 727-733. | Participants were not athlete |
| Fletcher, I. M., & Anness, R. (2007). The acute effects of combined static and dynamic stretch protocols on fifty-meter sprint performance in track-and-field athletes. *Journal of strength and conditioning research*, *21*(3), 784–787. | Participants were not athletes from the specific sports listed in the inclusion criteria |
| Kistler, B. M., Walsh, M. S., Horn, T. S., & Cox, R. H. (2010). The acute effects of static stretching on the sprint performance of collegiate men in the 60- and 100-m dash after a dynamic warm-up. *Journal of strength and conditioning research*, *24*(9), 2280–2284. | Participants were not athletes from the specific sports listed in the inclusion criteria |
| Favero, J. P., Midgley, A. W., & Bentley, D. J. (2009). Effects of an acute bout of static stretching on 40 m sprint performance: influence of baseline flexibility. *Research in sports medicine*, *17*(1), 50-60. | Participants were not athletes from the specific sports listed in the inclusion criteria |
| La Torre, A., Castagna, C., Gervasoni, E., Cè, E., Rampichini, S., Ferrarin, M., & Merati, G. (2010). Acute effects of static stretching on squat jump performance at different knee starting angles. The Journal of Strength & Conditioning Research, 24(3), 687-694. | The participants included athletes from both track and field and soccer, representing both recreational and competitive levels |
| Lowery, R. P., Joy, J. M., Brown, L. E., de Souza, E. O., Wistocki, D. R., Davis, G. S., ... & Wilson, J. M. (2014). Effects of static stretching on 1-mile uphill run performance. The *Journal of Strength & Conditioning Research*, 28(1), 161-167. | Participants were not athletes from the specific sports listed in the inclusion criteria |
| **Intervention** | |
| Silva, D. S., Boullosa, D., Pereira, E. V., Alves, M. D., Fernandes, M. S., Aidar, F., ... & de Souza, R. F. (2023). Does the sequence of plyometric and dynamic stretching exercises influence subsequent sprint performance? A randomized crossover intervention study. *Biology of Sport*, *41*(2), 13-18. | The stretching protocol was combined with the plyometric protocol |
| Cuenca-Fernández, F., Konrad, A., & Tilp, M. (2026). Stretching and potentiation for performance optimization: effects on upper limbs in competitive swimmers. *European journal of applied physiology*, 10.1007/s00421-026-06133-9. Advance online publication. | The stretching protocol was combined with aerobic and resistance protocols |
| Wyon, M., Felton, L., & Galloway, S. (2009). A comparison of two stretching modalities on lower-limb range of motion measurements in recreational dancers. *The Journal of Strength & Conditioning Research*, *23*(7), 2144-2148. | The study evaluated chronic effects |
| Zhang, W., & Bai, N. (2022). The role of functional dynamic stretching training in dance sports. *Revista Brasileira de Medicina do Esporte*, *28*, 837-839. | The study evaluated chronic effects |
| Lima, C. D., Brown, L. E., Wong, M. A., Leyva, W. D., Pinto, R. S., Cadore, E. L., & Ruas, C. V. (2016). Acute effects of static vs. ballistic stretching on strength and muscular fatigue between ballet dancers and resistance-trained women. *The Journal of Strength & Conditioning Research*, *30*(11), 3220-3227. | The study evaluated chronic effects |
| Kafkas, A., Eken, Ö., Kurt, C., & Kafkas, M. E. (2019). The effects of different stretching and warm-up exercise protocols on 50-meter swimming performance in sub-elite women swimmers. *Isokinetics and Exercise Science*, *27*(4), 289-297. | The study evaluated chronic effects |
| Özengin, N., Yıldırım, N. Ü., Baltacı, G., & Masiulis, N. (2011). Acute effects of different stretching durations on vertical jump performance in rhythmic gymnasts. *Baltic Journal of Sport and Health Sciences*, 3(82). | The study evaluated chronic effects |
| **Comparison** | |
| Nelson, A. G., Driscoll, N. M., Landin, D. K., Young, M. A., & Schexnayder, I. C. (2005). Acute effects of passive muscle stretching on sprint performance. *Journal of sports sciences,*23(5), 449-454. | Absence of pre-and post-comparisons |
| Siatras, T., Papadopoulos, G., Mameletzi, D., Gerodimos, V., & Kellis, S. (2003). Static and dynamic acute stretching effect on gymnasts’ speed in vaulting. *Pediatric Exercise Science*, 15(4), 383-391. | Absence of pre-and post-comparisons |
| Silva, G. C. E., Silveira, A., Novaes, J., Di Masi, F., Conceicao, M., & Dantas, E. (2014). Acute effects of static and proprioceptive neuromuscular facilitation stretching on sprint performance in male swimmers. *Med Sport*, *67*, 119-28. | Absence of pre-and post-comparisons |
| Winchester, J. B., Nelson, A. G., Landin, D., Young, M. A., & Schexnayder, I. C. (2008). Static stretching impairs sprint performance in collegiate track and field athletes. *The Journal of Strength & Conditioning Research*, *22*(1), 13-19. | Absence of pre-and post-comparisons |

**Supplementary Table S3: Kappa value for study selection**

There were 23 studies evaluated by two reviewers. Both reviewers agreed completely on all of their decisions.

**Contingency Table**

|  | Reviewer 2: Yes | Reviewer 2: No | Total for Reviewer 1 |
| --- | --- | --- | --- |
| Reviewer 1: Yes | 23 | 0 | 23 |
| Reviewer 1: No | 0 | 0 | 0 |
| Total for Reviewer 2 | 23 | 0 | 23 |

**Observed Agreement (P_o_):** The observed agreement is the proportion of studies that both reviewers agreed upon, which in this case was 100% agreement.

P_o_= $\frac{23+0}{23+0+0+0}$= $\frac{23}{23}$= 1

**Expected Agreement (Pe):** Since both reviewers agreed on all studies, we expected that the observed agreement was the same as the expected agreement.

**Cohen’s Kappa (*κ*):**

*K*= $\frac{P_{o}-P_{e}}{1-P_{e}}$= $\frac{1-1}{1-1}$= $\frac{0}{0}$

Since the denominator is 0, this calculation results in undefined. However, in practice, when perfect agreement is achieved, the Kappa value is considered 1 by definition.

**Supplementary Table 4: Modified Downs and Black**

|  | **Modified Downs and Black checklist item** | | | | | | | | | | | | | | | **Total**  **score** | **Quality** |
| --- | --- | --- | --- | --- | --- | --- | --- | --- | --- | --- | --- | --- | --- | --- | --- | --- | --- |
| **Study** | **Reporting** | | | | | | | **External validity** | | **Internal validity (bias)** | | | **Internal Validity (confounding)** | | |  |  |
|  | **1** | **2** | **3** | **5** | **6** | **7** | **10** | **11** | **12** | **16** | **18** | **20** | **21** | **22** | **25** |  |  |
| 1. Sands et al. (2008) | 1 | 1 | 1 | 0 | 1 | 1 | 1 | 0 | 0 | 1 | 1 | 1 | 1 | 0 | 0 | 10 | Low |
| 1. Di Cagno et al. (2010) | 1 | 1 | 1 | 0 | 1 | 1 | 1 | 0 | 0 | 1 | 1 | 1 | 1 | 0 | 0 | 11 | Moderate |
| 1. Rubini et al. (2011) | 1 | 1 | 1 | 0 | 1 | 1 | 1 | 0 | 0 | 1 | 1 | 1 | 1 | 0 | 0 | 10 | Low |
| 1. Agopyan et al. (2012) | 1 | 1 | 1 | 1 | 1 | 1 | 1 | 0 | 0 | 1 | 1 | 1 | 1 | 0 | 0 | 11 | Moderate |
| 1. Williams et al. (2012) | 1 | 1 | 1 | 0 | 1 | 1 | 1 | 0 | 0 | 1 | 1 | 1 | 1 | 0 | 0 | 10 | Low |
| 1. Agopyan et al. (2013) | 1 | 1 | 1 | 0 | 1 | 1 | 1 | 0 | 0 | 1 | 1 | 1 | 1 | 0 | 0 | 10 | Low |
| 1. Morrin & Redding (2013) | 1 | 1 | 1 | 0 | 1 | 1 | 1 | 0 | 0 | 1 | 1 | 1 | 1 | 0 | 0 | 10 | Low |
| 1. Cengiz et al. (2014) | 1 | 1 | 1 | 0 | 1 | 1 | 1 | 0 | 0 | 1 | 1 | 1 | 1 | 0 | 0 | 10 | Low |
| 1. Costa E Silva et al. (2014) | 1 | 1 | 1 | 0 | 1 | 1 | 0 | 0 | 0 | 1 | 1 | 1 | 1 | 0 | 0 | 9 | Low |
| 1. Donti et al. (2014) | 1 | 1 | 1 | 0 | 1 | 1 | 1 | 0 | 0 | 1 | 1 | 1 | 1 | 0 | 0 | 10 | Low |
| 1. Bogdanis et al. (2017) | 1 | 1 | 1 | 0 | 1 | 1 | 1 | 0 | 0 | 1 | 1 | 1 | 1 | 0 | 0 | 10 | Low |
| 1. Johnson et al. (2018) | 1 | 1 | 1 | 0 | 1 | 1 | 1 | 0 | 0 | 1 | 1 | 1 | 1 | 0 | 0 | 10 | Low |
| 1. Papia et al. (2018) | 1 | 1 | 1 | 0 | 1 | 1 | 1 | 0 | 0 | 1 | 1 | 1 | 1 | 0 | 0 | 10 | Low |
| 1. De la Cruz-Torres et al. (2019) | 1 | 1 | 1 | 2 | 1 | 1 | 1 | 0 | 0 | 1 | 1 | 1 | 1 | 1 | 0 | 13 | High |
| 1. Dallas et al. (2019) | 1 | 1 | 1 | 0 | 1 | 1 | 0 | 0 | 0 | 1 | 1 | 1 | 1 | 0 | 0 | 9 | Low |
| 1. Balcı et al. (2020) | 1 | 1 | 1 | 2 | 1 | 1 | 1 | 0 | 0 | 1 | 1 | 1 | 1 | 0 | 1 | 13 | High |
| 1. Pessali-Marques et al. (2020) | 1 | 1 | 1 | 0 | 1 | 1 | 1 | 0 | 0 | 1 | 1 | 1 | 1 | 0 | 0 | 10 | Low |
| 1. Dierick et al. (2021) | 1 | 1 | 1 | 0 | 1 | 1 | 1 | 0 | 0 | 1 | 1 | 1 | 1 | 0 | 0 | 10 | Low |
| 1. Ari (2021) | 1 | 1 | 1 | 0 | 1 | 1 | 1 | 0 | 0 | 1 | 1 | 1 | 1 | 0 | 0 | 10 | Low |
| 1. Kurt et al. (2024) | 1 | 1 | 1 | 0 | 1 | 1 | 1 | 0 | 0 | 1 | 1 | 1 | 1 | 1 | 0 | 11 | Moderate |
| 1. Durukan et al. (2025) | 1 | 1 | 1 | 1 | 1 | 1 | 1 | 0 | 0 | 1 | 1 | 1 | 1 | 1 | 0 | 12 | Moderate |
| 1. Jochum et al. (2025) | 1 | 1 | 1 | 1 | 1 | 1 | 1 | 0 | 0 | 1 | 1 | 1 | 1 | 1 | 1 | 13 | High |
| 1. Yan et al. (2025) | 1 | 1 | 1 | 1 | 1 | 1 | 1 | 0 | 0 | 1 | 1 | 1 | 1 | 1 | 1 | 13 | High |
| **Overall score** |  | | | | | | | | | | | | | | | **10.65** |  |

**Descriptions:** Downs and Black checklist item descriptions: 1, hypothesis/aims/objectives reported; 2, main outcome measures reported; 3,participant characteristics reported; 5, principal confounders reported; 6, main findings reported; 7, variability in main outcomes reported; 10, probability values reported; 11, source population representative of entire population; 12, study population representative of source population; 16, no retrospective subgroup analysis; 18, statistical tests are appropriate; 20, outcome measures are valid and reliable; 21, recruitment of study groups from same population; 22, recruitment of participants over same period; 25, adjustment for confounding variables in main analysis. Overall quality: Low, ≤10; Moderate, 11 or 12; High, ≥13.

**The modified version of the Downs & Black checklist (15-item)**

| **Item** | **Yes** | **Unable to determine** | **No** |
| --- | --- | --- | --- |
| 1. Is the hypothesis/aim/objective of the study clearly described? | 1 |  | 0 |
| 2. Are the main outcomes to be measured clearly described in the Introduction or Methods section? | 1 |  | 0 |
| 3. Are the characteristics of the patients included in the study clearly described? | 1 |  | 0 |
| 5. Are the distributions of principal confounders in each group of subjects to be compared clearly described? | 2 | Partially 1 | 0 |
| 6. Are the main findings of the study clearly described? | 1 |  | 0 |
| 7. Does the study provide estimates of the random variability in the data for the main outcomes? | 1 |  | 0 |
| 10. Have actual probability values been reported (e.g. 0.035 rather than <0.05) for the main outcomes except where the probability value is less than 0.001? | 1 |  | 0 |
| *External Validity* |  |  |  |
| 11. Were the subjects asked to participate in the study representative of the entire population from which they were recruited? | 1 | 0 | 0 |
| 12. Were those subjects who were prepared to participate representative of the entire population from which they were recruited? | 1 | 0 | 0 |
| *Internal Validity - Bias* |  |  |  |
| 16. If any of the results of the study were based on “data dredging”, was this made clear? | 1 | 0 | 0 |
| 18. Were the statistical tests used to assess the main outcomes appropriate? | 1 | 0 | 0 |
| 20. Were the main outcome measures used accurate (valid and reliable)? | 1 | 0 | 0 |
| *Internal Validity – Confounding (selection bias)* |  |  |  |
| 21. Were the subjects in different intervention groups (trials and cohort studies) or were the cases and controls (case-control studies) recruited from the same population? | 1 | 0 | 0 |
| 22. Were study subjects in different intervention groups (trials and cohort studies) or were the cases and controls (case-control studies) recruited over the same period of time? | 1 | 0 | 0 |
| 25. Was there adequate adjustment for confounding in the analyses from which the main findings were drawn? | 1 | 0 | 0 |
